# Variation in Size and Growth of the Great Scallop *Pecten maximus* along a Latitudinal Gradient

**DOI:** 10.1371/journal.pone.0037717

**Published:** 2012-05-23

**Authors:** Laurent Chauvaud, Yann Patry, Aurélie Jolivet, Emmanuelle Cam, Clement Le Goff, Øivind Strand, Grégory Charrier, Julien Thébault, Pascal Lazure, Karl Gotthard, Jacques Clavier

**Affiliations:** 1 Université de Bretagne Occidentale; Institut Universitaire Européen de la Mer, Laboratoire des Sciences de L'Environnement Marin (UMR CNRS 6539), Technopôle Brest Iroise, Plouzané, France; 2 UMR CNRS 5175, Biométrie et Biologie des Populations. Centre d'Écologie Fonctionnelle et Évolutive, Montpellier, France; 3 Laboratoire Évolution et Diversité Biologique, UMR CNRS 5174, Université Paul Sabatier, Toulouse III, Toulouse, France; 4 Institute of Marine Research, Bergen, Norway; 5 Department of Marine Ecology – Tjärnö, University of Gothenburg, Strömstad, Sweden; 6 IFREMER, Laboratoire d'Océanographie Spatiale (LOS) et Laboratoire Physique Hydrodynamique et Sedimentaire (PHYSED), Centre de Brest, Plouzané, France; 7 Department of Zoology, Stockholm University, Stockholm, Sweden; University of Bristol, United Kingdom

## Abstract

Understanding the relationship between growth and temperature will aid in the evaluation of thermal stress and threats to ectotherms in the context of anticipated climate changes. Most *Pecten maximus* scallops living at high latitudes in the northern hemisphere have a larger maximum body size than individuals further south, a common pattern among many ectotherms. We investigated differences in daily shell growth among scallop populations along the Northeast Atlantic coast from Spain to Norway. This study design allowed us to address precisely whether the asymptotic size observed along a latitudinal gradient, mainly defined by a temperature gradient, results from differences in annual or daily growth rates, or a difference in the length of the growing season. We found that low annual growth rates in northern populations are not due to low daily growth values, but to the smaller number of days available each year to achieve growth compared to the south. We documented a decrease in the annual number of growth days with age regardless of latitude. However, despite initially lower annual growth performances in terms of growing season length and growth rate, differences in asymptotic size as a function of latitude resulted from persistent annual growth performances in the north and sharp declines in the south. Our measurements of daily growth rates throughout life in a long-lived ectothermic species provide new insight into spatio-temporal variations in growth dynamics and growing season length that cannot be accounted for by classical growth models that only address asymptotic size and annual growth rate.

## Introduction

The study of latitudinal variation in organism size both within and between species has a long tradition, since Bergmann's work on mammals, describing the individual tendency to be larger in cold environments [1], [2], [3], [4], [5], [6], [7], [8], [9], [10], [11], [12], [13], [14]. This has been of interest because it may reflect important ecological interactions between the organisms and their environment, and because it may help in understanding the evolutionary dynamics of size and growth patterns in relation to latitudinal varying selection pressures. In more recent years, it is clear that the study of latitudinal variation has been prompted partly by the fact that thermal conditions vary with latitude and that it may be possible to explore this spatial variation to evaluate the expected effect of increased temperatures on both ecological and evolutionary processes. Given that the projections of global temperature increase is ranging from 1.8°C to 4°C from the 1980s to the end of the 21^st^ century [15], our ability to understand the relationship between growth patterns and temperature is important because global climate change will be a thermal challenge to most ectotherms [16]. However, the mechanisms responsible for body size variation over broad geographical scales in long-lived ectotherms have seldom been identified in the field over longer stretches of time.

The growth models commonly used to assess growth trajectories in populations, such as the logistic, Gompertz, or von Bertalanffy curves, are fitted at the population level and yield only an average representation of individual growth that does not account for variability among individuals. The popularity of these models likely is due to their ability to enable comparisons among populations based on a limited number of standard model parameter estimates [17]. In addition, information on the length of the growing season, maximum growth rate, or their variations over time is often missing. As a consequence, analysis of body size variation at the broad geographic scale is often based on overall, population-averaged comparisons of growth trajectories, which may mask differences in growth patterns among locations and environmental conditions.

Evidence for seasonal variation in growth in marine invertebrates comes for example from the bryozoan *Cellarinella watersi* Calvet [18], the sea urchin *Sterechinus neumayeri* (Meissner) [19], and the great scallop *Pecten maximus* (L.) [20], [21]. The capacity for growth within the same species or within taxonomically closely related species may vary inversely with the length of the growing season across a latitudinal gradient, thus compensating for environmental effects [2], [22], [23]. Since maintenance costs are related to an individual's size and volume [24], energy requirements increase each year with increasing size (growth-maintenance trade-off). The growth-reproduction trade-off may also require individuals to devote an increasing amount of resources to reproduction and, as a consequence, less resources to growth as they age. However, growth efficiency is greater at low temperatures because less energy is consumed for maintenance [25]. Hence, within a species, individuals may allocate resources to growth and reproduction differentially depending on thermal conditions (growth-reproduction and growth-defense trade-offs [26], [27]), which may lead to significant latitudinal variation in growth dynamics over life.

The great scallop *P. maximus* is distributed along the Northeast Atlantic coasts. Here we explore variation in growth patterns in this species along a latitudinal gradient using three main parameters: the maximum annual growth rate, the daily growth rate, and the length of the growing season. Our biological model and laboratory techniques [28] allow fine assessment of the growth dynamics of individual scallops on a daily basis throughout the lifespan of the organism, providing new insight into spatio-temporal changes in growth dynamics compared to traditional growth models such as the von Bertalanffy model. We address five hypotheses in this investigation: i) that asymptotic size varies with latitude, ii) that asymptotic size is negatively related to the annual growth rate, iii) that low annual growth rates reflect low daily growth rates or a combination of high daily growth rates and short growing season, iv) that the length of the growing season decreases with age, and v) that the decrease in the length of the growing season with age should be more rapid with lower latitudes.

## Methods

### Sampling

**Figure 1:**
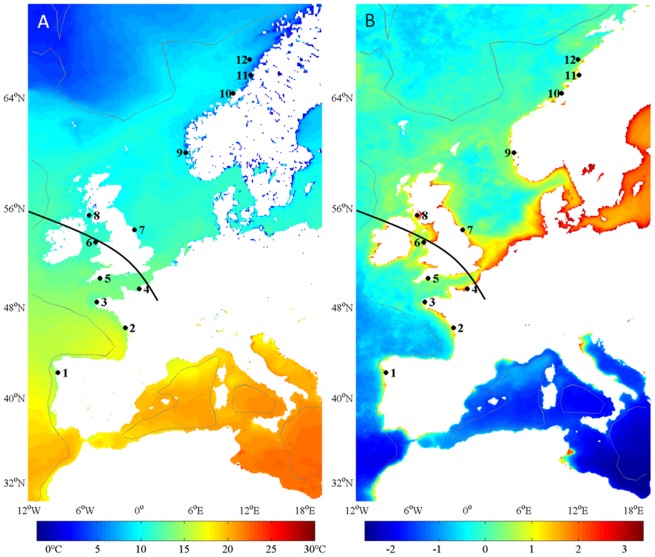
Characterization of the study area by (A) average annual temperature (°C) and (B) log-transformed chlorophyll *a* concentration (mg m^−3^). The dark line represents the general limit of southern species established by Forbes [55], and the dark circles correspond to the *P. maximus* populations sampled from (1) Vigo, (2) Ile de Re, (3) Bay of Brest, (4) Bay of Seine, (5) Plymouth, (6) Holyhead, (7) Scarborough, (8) Campbell Town, (9) Austevoll, (10) Bessaker, (11) Bronnoysund, and (12) Traena.

*P. maximus* individuals were sampled from 2000 to 2005 by dredging or scuba diving in 12 wild populations distributed along the Northeast Atlantic coast (Table 1, Figure 1). For facilitation of the identification of relationships between growth and latitude, the samples were collected at constant depth (15–20 m).

**Table 1:** Description of the 12 sampled stations.

Stations	Name	Latitude	Longitude	Annual Temp	Annual chloro	n (VB)	age max (VB)	n (DG)	age max (DG)
1	Vigo	42°23′N	8°71′W	15.28	4.84	71	7	11	4
2	Ile de Ré	46°20′N	1°40′W	15.18	3.98	51	6	15	3
3	Rade de Brest	48°23′N	4°28′W	13.23	3.14	60	6	32	4
4	Baie de Seine	49°50′N	0°19′W	13.00	7.37	52	6	29	3
5	Plymouth	50°20′N	4°08′W	13.49	2.34	30	6	18	4
6	Holyhead	53°03′N	4°42′W	11.37	2.98	34	9	14	4
7	Scarborough	54°19′N	0°06′E	10.51	4.03	51	7	17	5
8	Campbell town	55°26′N	5°31′W	10.42	9.31	50	7	27	4
9	Austevoll	60°06′N	5°10′E	7.92	6.54	13	7	38	6
10	Bessaker	64°15′N	10°19′E	8.46	6.28	33	9	15	6
11	Bronnoyusund	65°27′N	11°25′E	7.62	2.44	50	10	8	5
12	Traena	66°30′N	12°21′E	8.01	1.26	50	11	23	6

Main geographic characteristics of the study stations are detailed (latitude, longitude, annual average temperature and chlorophyll *a* concentrations) as the number of individuals used for estimating parameters of the von Bertalanffy growth model and used for estimating the mean growth trajectory and the maximum age observed in the two analyses.

### Estimating growth parameters

Age was determined by enumeration and interpretation of annuli, annual visible marks on the surface of shells [29], [30]. Individual dorso–ventral height at each age was obtained by back calculation, measuring the distance between the umbo and winter rings along the axis of maximum growth of the shell.

The specialized von Bertalanffy growth function was fitted to data from each sampling station according to the equation H_t_ = H_∞_×(1−e^k (t-to)^), where H_t_ represents the expected or average shell height (mm) at time t (yr), H_∞_ is the mean asymptotic shell height (mm), k is the Brody growth rate coefficient (yr^−1^), and t_o_ is the theoretical age (yr) at which shell height equals zero. We performed the joint estimation of H_∞_, k, and t_o_ and their confidence intervals by nonlinear fitting using a Marquardt algorithm on a sample of at least 30 individuals per station, except for station 9 (Austevoll; Table 1). The index of the overall growth performance (Φ′) was defined as the maximum growth rate (*i.e.* the growth rate at the inflexion point of the von Bertalanffy growth function), and was used to compare growth between population and species (for review, see [31]). The index was calculated from the von Bertalanffy parameters according to the Pauly and Munro [32] equation: Φ′ = log(k)+2log (0.1×H_∞_), where k is in year^−1^ and H_∞_ in mm.

### Acquisition of daily growth data

The construction of the bivalve carbonate skeleton results from successive accretion of material on the outer edge of the shell. In *P. maximus*, the formation of microstructures called “striae” occurs daily [28], [33], [34]. For each individual, we estimated the daily growth rate by measuring the distance between two consecutive daily growth striae from the earliest detectable one to the outer edge of the shell. The daily growth patterns of each individual's flat valve were examined on images acquired using a high-resolution video camera (Sony DFW-X700) and analyzed with image analysis software (Visilog ®, Noesis, see [28] for additional information).

To build the mean growth trajectories of the studied populations, we performed a synchronization procedure between the individual growth trajectories from a single cohort, with the number of included individuals varying from 8 in Bronnoysund to 38 in Austevoll (Table 1). As the growth of *P. maximus* stops in winter [28], [33], [34], the synchronization was performed for each year of growth by minimizing the sum of the differences between individual series considered two-by-two. This approach allowed us to obtain a mean daily growth rate for each age class and for each sampled population. The series of growth values were ordered following the position of striae along the growth axis from the umbo to the outer edge. Thus, the succession of growth striae describing a “time” axis (days of growth) provided a continuous representation of successive growth years (truncation of winter episodes without growth). By convention, the age class is the number of 1^st^ January days experienced by the individual.

The duration of the growth phase and the maximum annual growth rate permitted an initial characterization of seasonal shell growth. From the mean growth trajectories of the studied populations, we obtained the maximum annual growth rate according to the average distance corresponding to the 10 widest successive inter-striae. The number of growth days was obtained by counting the striae between two successive minima (two winters). A linear model allowed assessment of the relationship between the number of days of annual growth and the age of individuals. The slope of this model, denoted by “Ω,” is an estimator of the decrease in the number of growth days with age, constituting an additional way to compare populations.

### Acquisition of environmental data

We collected sea surface temperature and chlorophyll *a* concentration (mg m^−3^) measurements along the latitudinal gradient from the satellite sensor MODIS (Moderate Resolution Imaging Spectroradiometer) available at http://oceancolor.gsfc.nasa.gov/. We used the archive corresponding to the seasonal climatology acquired between 2003 and 2010 at 9-km resolution. For each sampling station, we calculated the average annual temperature and chlorophyll *a* concentrations from the whole climatology (2003–2010) corresponding to a rectangle of 1° latitude by 1° longitude centered on each point (Table 1).

### Statistical Analysis

As in Heilmayer et al. [35], we used an Arrhenius model to describe the effects of temperature on the index of the overall growth performance (Φ′) of *P. maximus*, defined by the equation: ln (Φ′) = α×1/T+β, where T is the absolute temperature (in K), α is the slope corresponding to the Arrhenius activation energy, and β is the constant. Pearson correlation was used to explore relationships between latitude (in units of decimal degrees) and the two environmental factors (sea surface temperature and chlorophyll *a* concentration). ANOVA was also performed between the growth parameters of all studied stations. Linear regressions were generally used to establish relationships between temperature and growth parameters. The studentized residuals were analyzed and compared to the *t*-test value for outlier detection. In case of discontinuities, a model with two regressions was performed and subjected to single linear regression by the Chow test (test of the sum of squared residuals).

## Results

### Environment

The annual averages of temperature and chlorophyll *a* concentrations are presented in Figure 1. The latitudinal gradient is mainly described by a negative correlation with the temperature (R^2^ = 0.93, F = 114, degrees of freedom model/errors: dfm/dfe = 1/10, P<0.001) and is not linked to the average chlorophyll *a* concentration (R^2^ = 0.06, F = 0.66, dfm/dfe = 1/10, P = 0.44). The average chlorophyll *a* concentration does not behave as a discriminatory parameter along the studied latitudinal gradient; relationships between growth parameters and the latitude gradient have thus been analyzed according to temperature.

### Size variations

**Figure 2:**
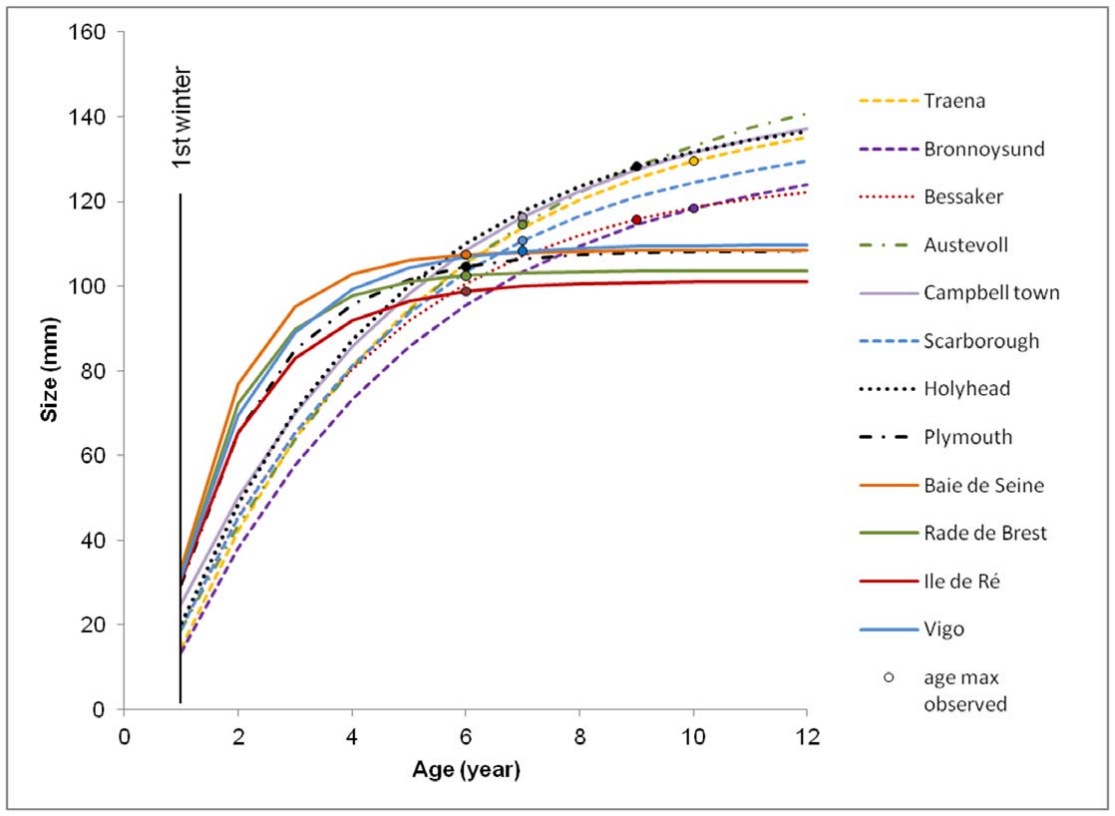
Von Bertalanffy growth curves obtained for the 12 studied populations. By convention, the age class is the number of 1^st^ January days experienced by the individual. The age maximum observed was specified for each population varying from 6 to 10 years (circles).

**Figure 3:**
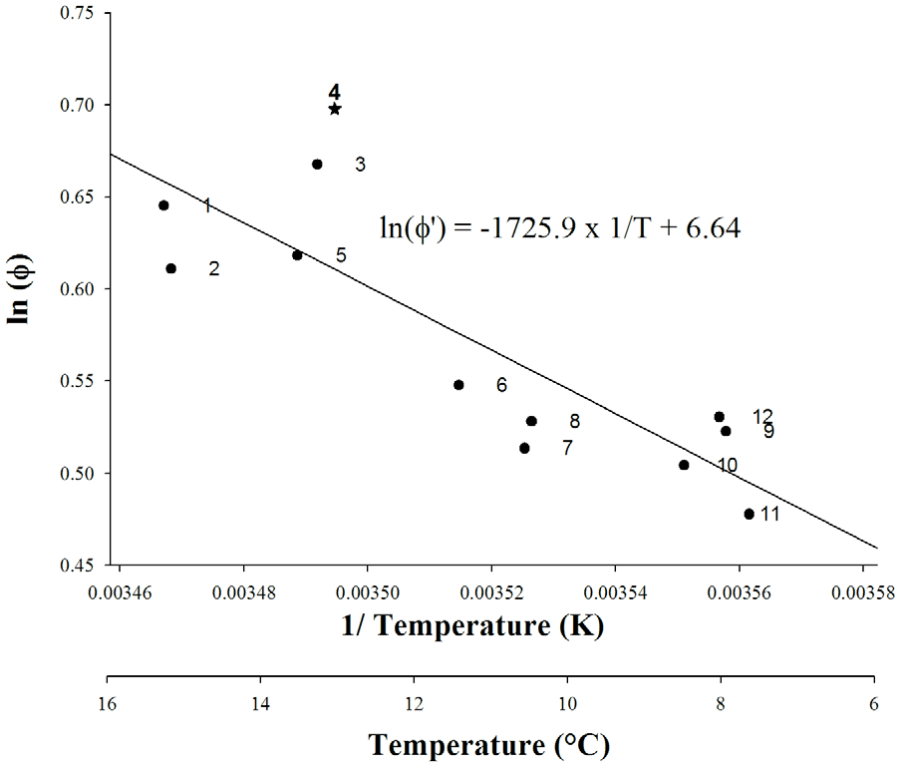
Relationships between the mean annual sea surface temperature and the growth performance index (Φ′). This index issued from the Arrhenius model was given for populations sampled in (1) Vigo, (2) Ile de Re, (3) Bay of Brest, (4) Bay of Seine, (5) Plymouth, (6) Holyhead, (7) Scarborough, (8) Campbell Town, (9) Austevoll, (10) Bessaker, (11) Bronnoysund, and (12) Traena.

Annual growth measurements were acquired on more than 30 individuals per population except for Austevoll (n = 13, Table 1). The maximum observed age was of 6 to 7 years for the southern populations (Figure 2, stations 1–5) and 7 to 10 years for the northern populations (Figure 2, stations 6–12). The von Bertalanffy growth model was fitted to these measurements and given on Figure 2. A temperature-size gradient was clearly identifiable within the *P. maximus* distribution area with a positive correlation for the first five classes (Figure 2; class 1, R^2^ = 0.83, F = 47.7, dfm/dfe = 1/10, P<0.001; class 5, R^2^ = 0.58, F = 13.7, dfm/dfe = 1/10, P = 0.004). Lower annual growth characterized shells from the northern stations. This growth differential decreased with age, and size differences between populations were no longer significant after six winters (Figure 2; class 6, R^2^ = 0.08, F = 0.85, dfm/dfe = 1/10, P = 0.38). On the contrary for the classes superior to seven, a negative correlation with the temperature was observed (Figure 2; H_∞_, R^2^ = 0.66, F = 19.6, dfm/dfe = 1/10, P = 0.001). The resulting index growth performance (Φ′) varied from 1.61 in Bronnoysund to 2.01 in the Bay of Seine (Table 2). In the Arrhenius model, Φ′ was positively correlated with temperature (Figure 3; R^2^ = 0.70, F = 22.8, dfm/dfe = 1/10, P<0.001). *P. maximus* growing in the Nordic stations thus display a slower growth rate than individuals in the southern stations, but northern individuals achieve a higher asymptotic length. Analysis of the studentized residuals revealed the Bay of Seine station as an outlier point (Figure 3; station 4, t = 2.47, df = 10, P = 0.033).

**Table 2:** Summary growth data.

Stations	Name	L_∞_ (mm)	k (year^−1^)	T_0_ (year)	R^2^	Φ′	MDG
1	Vigo	109.7 [107.6 111.8]	0.67 [0.62 0.72]	0.50 [0.46 0.54]	0.96	1.91	257.9±5.1
2	Ile de Re	101.1 [98.4 103.8]	0.68 [0.61 0.75]	0.47 [0.41 0.53]	0.95	1.84	217.7±2.1
3	Bay of Brest	103.6 [101.3 105.9]	0.83 [0.76 0.90]	0.56 [0.52 0.60]	0.97	1.95	241.0±1.6
4	Bay of Seine	108.4 [104.7 112.0]	0.87 [0.76 0.97]	0.58 [0.53 0.63]	0.95	2.01	260.9±1.3
5	Plymouth	108.4 [102.9 113.8]	0.61 [0.52 0.71]	0.48 [0.40 0.56]	0.96	1.86	223.1±2.4
6	Holyhead	143.6 [136.3 150.9]	0.26 [0.23 0.29]	0.41 [0.32 0.49]	0.97	1.73	261.3±2.1
7	Scarborough	137.0 [126.8 147.2]	0.25 [0.21 0.29]	0.40 [0.31 0.50]	0.95	1.67	273.3±5.6
8	Campbell town	146.9 [131.9 161.8]	0.23 [0.18 0.27]	0.19 [0.07 0.31]	0.95	1.70	264.8±3.4
9	Austevoll	155.9 [126.5 185.2]	0.20 [0.13 0.28]	0.36 [0.16 0.56]	0.95	1.69	210.6±1
10	Bessaker	127.2 [118.5 135.9]	0.28 [0.24 0.33]	0.42 [0.30 0.55]	0.94	1.66	235.6±2.9
11	Bronnoysund	133.5 [128.6 138.4]	0.23 [0.21 0.25]	0.54 [0.46 0.61]	0.97	1.61	240.6±5.8
12	Traena	144.5 [139.3 149.8]	0.24 [0.22 0.26]	0.56 [0.49 0.63]	0.97	1.70	261.0±1.8

Von Bertalanffy growth parameters and index of growth performance (Φ′) were fitted from growth data of each study station (in brackets, the limits of the asymptotic 95% confidence interval). Maximum daily growth, MDG (in µm d^−1^), was averaged on the ten highest successive increments (± standard error).

### Seasonal variations in growth parameters

Daily growth was measured along three to six years following population (Table 1). This number of class differed from what observed for the von Bertalanffy models because from a certain age the winter rings are readable unlike daily marks.

**Figure 4:**
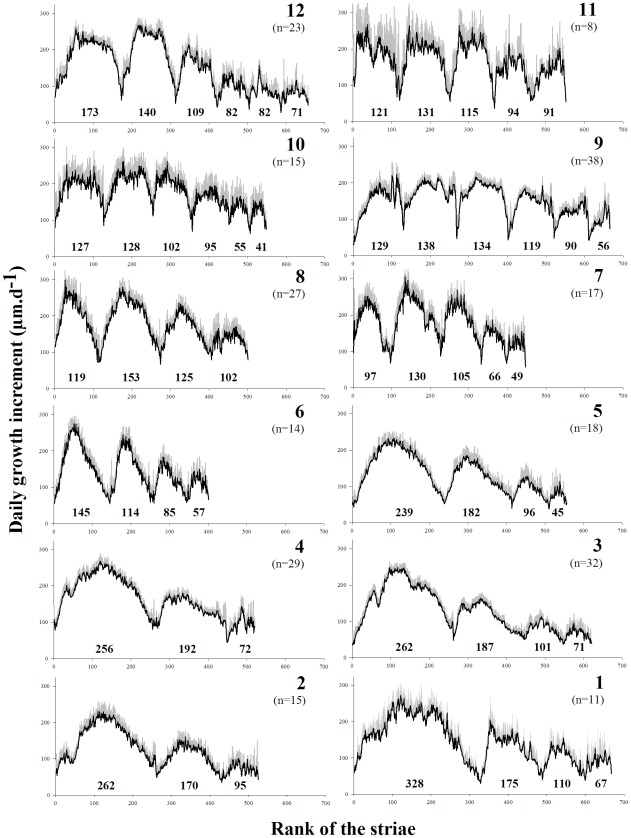
Variation in mean daily shell growth throughout life (black line) for each sampled population. Gray vertical bars represent the 95% confidence interval of each mean value. The numbers in each graph indicate the annual number of growth days. (1) Vigo, (2) Ile de Re, (3) Bay of Brest, (4) Bay of Seine, (5) Plymouth, (6) Holyhead, (7) Scarborough, (8) Campbell Town, (9) Austevoll, (10) Bessaker, (11) Bronnoysund, and (12) Traena.

Shell growth exhibited a strong seasonal cycle at all sites (Figure 4) that included a slowdown before the winter stop followed by relatively rapid spring and summer growth (≤50 µm d^−1^). The maximum daily growth rate significantly differed among populations (Table 2; one-way ANOVA, F = 38, dfm/dfe = 1/108, P<0.001) and ranged from 210 µm d^−1^ (standard error ±1) in Austevoll to 273 µm d^−1^±5.6 in Scarborough. However, the maximum daily growth rate was not correlated with temperature (R^2^<0.001, F = 0.006, dfm/dfe = 1/10, P = 0.94).

**Figure 5:**
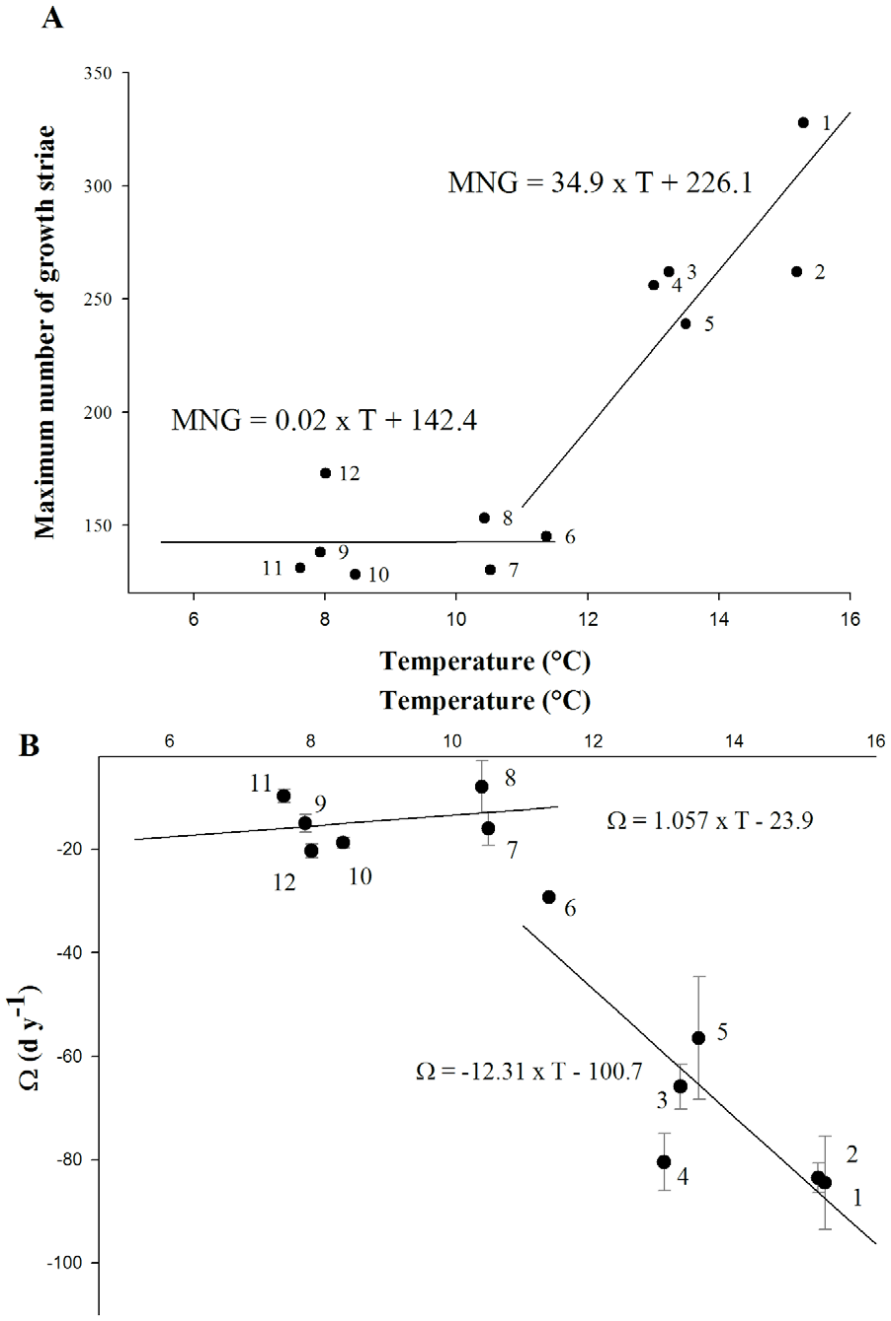
Relationships between the mean annual sea surface temperature and the daily growth parameters. This is shown for A) the maximum numbers of growth days (MNG); and B) the variation in the number of growth days with increasing age (Ω in d y^−1^) for populations sampled in (1) Vigo, (2) Ile de Re, (3) Bay of Brest, (4) Bay of Seine, (5) Plymouth, (6) Holyhead, (7) Scarborough, (8) Campbell Town, (9) Austevoll, (10) Bessaker, (11) Bronnoysund, and (12) Traena.

The maximum number of growth striae between two consecutive winters varied considerably among populations (Figure 4). The longest growth period occurred between the first and second winter in the southern stations, from Vigo to Plymouth, in contrast to the scallops sampled from Holyhead to Traena that experienced the maximal number of growth days between the second and third winters. This maximum number of growth striae was compared to the temperature, distinguishing two groups with a breakpoint at the Holyhead station (Figure 5A; Chow test, F = 9.9, dfm/dfe = 1/10, P = 0.007). For the southern stations, the maximum number of growth striae was negatively correlated with temperature (R^2^ = 0.75, F = 12.1, dfm/dfe = 1/4, P = 0.02); for the northern stations, no correlation with temperature was detected (R^2^<10^−6^, F<0.01, dfm/dfe = 1/5, P = 0.86).

### Multiyear growth variations

The length of the growth season decreased with *P. maximus* age, a phenomenon that was common to all populations but varied in intensity along the latitudinal gradient and was more substantial in the south (Figure 4). Ω varied among populations (Figure 5B), allowing us to sort populations into two geographical groups (Chow test, F = 8.5, dfm/dfe = 1/10, P = 0.011). The Nordic shells (stations 7–12) maintained an annual growth period close to that observed between the second and third winters (Figure 5B; R^2^ = 0.078, F = 0.34, dfm/dfe = 1/4, P = 0.59), whereas the southern populations (stations 1–6) exhibited drastic decreases every year as the individuals aged (Figure 5B; R^2^ = 0.71, F = 10, dfm/dfe = 1/4, P = 0.03).

## Discussion

Our observations are consistent with the hypothesis that the maximal size of *P. maximus* varies with latitude [6], a prerequisite for studies of spatio-temporal variation in growth trajectories. Moreover, our study populations conformed to a pattern that has been described in many species of ectotherms (larger asymptotic size at higher latitude in the northern hemisphere) but is not universal (e.g. [36], [37]).

### Relationships among annual and daily growth rate, length of growing season, and latitude

Our observations are also consistent with the hypothesis that asymptotic size is negatively related to annual growth rate. However, our measurements of daily shell growth (Figure 4) show that asymptotic size should be considered as the product of growth rate and growing season length [4], [13], [38], [39]. Annual growth rate is not sufficient to explain the size variations observed at broad geographic scales [40]. Like many marine ectotherms such as *Chionista fluctifraga* (G.B. Sowerby II) [41], *Conus tortilis* Conrad [42], and *Pinna nobilis* L. [43], *P. maximus* does not grow during the entire year but stops growing when environmental conditions become unfavorable (i.e. low temperature and low food availability, [20], [21], Figure 4). Temperature is typically suggested to be the main factor responsible for winter inactivity [44], [45], mainly because of its direct effect on the rates of biochemical reactions and its indirect effect on other physical environmental parameters (see [35] for a pectinid review, [46]).

The description of growth trajectory based on the daily shell growth increment from the overall growth performance index (Φ′) highlights a strong relationship with temperature. Previous worldwide comparisons indicated that growth performance increases with decreasing latitude [47]; this study has demonstrated a relationship with increasing temperature (Figure 3). Nevertheless, this index does not allow northern and southern subpopulations to be differentiated. Only the population sampled in the Bay of Seine exhibited higher growth performances (Figure 3) that may be related to the high productivity (Table 1) and the particular biogeography of the English Channel in terms of temperature, food, and currents [48], [49], [50].

The methodology of the present study, however, provides evidence that low annual growth rates (such as in northern populations) are not typically due to low daily growth values; rather, the relevant factor is the smaller number of days available each year to achieve this growth in the north as compared to the south. This is a novel result that would not have been possible if we had tried to explore the growth dynamics using only the body size of subsequent cohorts. We content that for a more complete understanding of intraspecific variation in size and growth patterns at a broad geographic scale it is important to characterize both the growth rate and the duration of growth.

### Decrease in length of growing season with age

Our observations support the notion that the annual number of growth days decreases with age in *P. maximus*, which is consistent with the hypothesis of trade-offs between growth and reproduction or growth and defense [26], [27], [51], [52], [53]. Changes in an organism's energy requirements over its lifetime may explain the decrease in the length of the annual growth season over life. Since metabolism costs, including maintenance, growth and production of gametes, depend on the individual's volume [24], energy requirements increase each year with size. In a seasonal environment, the date of growth restart indicates that food availability is sufficient to cover basal metabolic requirements and to allocate energy excess to growth. As individuals age, the energy “threshold” thus increases, resulting in a reduced period of annual growth.

An exception is the observed longer growing season in the third year of life (between the second and third winters) in northern populations (stations 7 to 11), in contrast to the hypothesized time period between the first and second winters (Figure 4). The small size of northern individuals at the beginning of the first winter may be associated with restricted energy reserves, which are depleted before spring. When food becomes available again, energy may be allocated to maintenance before starting shell growth. Since the reserves may be more important at the beginning of the second winter, this preliminary phase of allocation to maintenance may no longer be necessary or may be shorter, permitting a longer growing season the following year in northern populations.

### Higher growth efficiency at low temperature

Our last prediction was that the decrease in the length of the growing season with age should be more rapid at lower latitude. Two elements of our study support this hypothesis: i) the linear relationships between descriptors of growth (Ω, loss of growth days with age) and mean annual temperature at a given latitude (Figure 5), and ii) the gradual loss of proportionality along the gradient between the quantity of calcite precipitated and shell size (not illustrated here, [54]). This prediction can be explained by the fact that at lower latitude, individuals allocated more resources to maintenance. Indeed, although not considered explicitly in the original form of the von Bertalanffy model, the temperature is an important factor of the environment impacting the metabolic processes involved in the model (production/dissipation of tissues). This results, in the present study, in the positive correlation between the index of overall growth performance Φ′, coefficient calculated from the von Bertalanffy parameters (H_∞_, k), and the mean sea surface temperature. By observing pectinid bivalves of various species living under contrasting environment, Heilmayer et al. [35] accumulated strong empirical evidence that lower metabolic rate, a measure of the energy consumed by vital functions including maintenance and production of gametes, reduces energy costs of maintenance. That allows allocation of a larger fraction of metabolic energy to growth enhancing levels of growth performance and efficiency at lower temperatures.

However, this first interpretation seems to hide a shift in growth characteristics on either side of the British Isles. With the exception of the maximum daily growth rate, all growth parameters displayed an abrupt variation crossing the channel, in particular at the Holyhead station (Figures 3,5); otherwise, around the coasts of Britain and Ireland, many Northeast Atlantic continental-shelf species reach their northern or southern limits. The first description of the distributional limits of certain benthic species [55] included a delineation of the “general limit of southern types” (Figure 1). In the Northeast Atlantic Ocean, the Ushant Sea (“mer d'Iroise”) is as a biogeographical transition zone between the temperate and cold-temperate marine assemblages, with the Lusitanian province in the south and Boreal province in the north [56], [57]. As with other benthic invertebrates [58], the biogeographical distribution of *P. maximus* depends partly on larval transport and recruitment success, and its connectivity between south Brittany and the western English Channel populations has been reported to be low. Ayata et al. [48] failed to detect connectivity from the western English Channel to the Bay of Biscay in their model runs.

Otherwise, *P. maximus* belongs to present-day communities of the boreal-temperate region around the British Isles. We hypothesize that the metapopulation includes a subpopulation that survived in a northern glacial refuge (Pleistocene glacial maxima) and a subpopulation that returned from temperate regions following isotherm movements during interglacial periods. The phenomenon of the “Ushant Sea acting as a partly-permeable one-way barrier for connectivity (northwards water exchanges are scarce, whereas southwards larval exchanges are unlikely)” [48] should permit the maintenance of two contrasting growth trajectories in *P. maximus*. Past glacial history, ecological selection, and connectivity may together have produced two *P. maximus* populations with differential growth traits.

### Origin of intraspecific growth variations: phenotypic plasticity or directional selection?

Intraspecific variation in growth observed in species with wide geographical distributions is often assumed to reflect the adaptation of populations to local environmental conditions [59]. In the case of *P. maximus*, the ability of northern individuals to maintain a similar number of growth days in the first year of life and in subsequent years may reflect adaptation to the cooler environment. Indeed, a selective pressure favoring individuals with high growth potential in northern areas, where the growing season is short and temperatures are low, has been demonstrated along latitudinal gradients in marine fishes [5], [60]. However, molecular studies of the population structure of *P. maximus* along the Atlantic coast have revealed a very low genetic divergence between the populations of the United Kingdom, Norway, and France [61], [62]. These studies of population genetics are nevertheless mostly based on neutral genetic markers, and thus typically reflect neutral evolutionary processes such as gene flow and genetic drift. Hence, it is possible that the traits studied here is under strong and differential selection that is upholding local genetic adaptation along the cline. To test this adaptive hypothesis, common garden experiments and quantitative genetic analysis are necessary [63], [64]. In the absence of this type of data we cannot presently evaluate to what degree the latitudinal cline in growth patterns observed in *P. maximus* is due to local adaptation [65].

Other hypotheses not requiring genetic differentiation may also account for these observations, such as the different effects of temperature on anabolism and catabolism that may lead to the very common observation of an increase in body size of ectothermic organisms in colder environments; i.e. the so called “temperature size rule” [66], [67], [68]. Such hypotheses involve phenotypic plasticity, which may itself be adaptive [69]. Species translocations performed by Buestel et al. [70] provide evidence of phenotypic plasticity in growth along the latitudinal gradient for *P. maximus*. Indeed, populations with different origins (Britain, Ireland, and Scotland) and different original growth trajectories exhibited similar growth when individuals were transferred to the same site (Bay of Brest). Hence, plasticity in growth rate and body size in response to environmental heterogeneity is clearly present in scallops. Many biological models support a countergradient variation [5], [71], [72], but whether this phenomenon is adaptive plasticity [73] remains to be addressed.

Body size has been extensively studied from a biogeographical perspective and forms the cornerstone of Bergmann's rule: a general trend of animal sizes to increase with latitude [74]. Here we demonstrated that the increase of body size with latitude characterized as Bergmann's rule persists in annual growth performances throughout life. We suggest that myriad environmental factors potentially disrupt the adaptive pattern in body size reflected in Bergmann's rule by degrading monotonous contrasts in growth characteristics across latitudes.
